# Time-resolved fluorescence based direct two-site apoA-I immunoassays and their clinical application in patients with suspected obstructive coronary artery disease

**DOI:** 10.3389/fcvm.2022.912578

**Published:** 2022-10-14

**Authors:** Priyanka Negi, Taina Heikkilä, Karoliina Vuorenpää, Emilia Tuunainen, Wail Nammas, Teemu Maaniitty, Juhani Knuuti, Jari Metso, Janita Lövgren, Matti Jauhiainen, Urpo Lamminmäki, Kim Pettersson, Antti Saraste

**Affiliations:** ^1^Department of Life Technologies/Biotechnology, University of Turku, Turku, Finland; ^2^Heart Center, Turku University Hospital and University of Turku, Turku, Finland; ^3^Turku PET Centre, Turku University Hospital, University of Turku, Turku, Finland; ^4^Minerva Foundation Institute for Medical Research, Biomedicum, Helsinki, Finland; ^5^National Institute for Health and Welfare, Genomics and Biobank Unit, Biomedicum 2U, Helsinki, Finland

**Keywords:** coronary artery disease, phage display, high-density lipoprotein, apolipoprotein A-I, immunoassay, single-chain variable fragment, coronary computed tomography angiography, positron emission tomography

## Abstract

**Objective:**

High-density lipoprotein (HDL) is a heterogeneous group of subpopulations differing in protein/lipid composition and in their anti-atherogenic function. There is a lack of assays that can target the functionality of HDL particles related to atherosclerosis. The objective of this study was to construct two-site apolipoprotein A-I (apoA-I) assays and to evaluate their clinical performance in patients with suspected obstructive coronary artery disease (CAD).

**Approach and results:**

Direct two-site apoA-I assays (named 109–121 and 110–525) were developed to identify the presence of apoA-I in the HDL of patients with CAD using apoA-I antibodies as a single-chain variable fragment fused with alkaline phosphatase. ApoA-I^109−121^ and apoA-I^110−525^ were measured in 197 patients undergoing coronary computed tomography angiography (CTA) and myocardial positron emission tomography perfusion imaging due to suspected obstructive CAD. Among patients not using lipid-lowering medication (LLM, *n* = 125), the level of apoA-I^110−525^ was higher in the presence than in the absence of coronary atherosclerosis [21.88 (15.89–27.44) mg/dl vs. 17.66 (13.38–24.48) mg/dl, *P* = 0.01)], whereas there was no difference in apoA-I^109−121^, HDL cholesterol, and apoA-I determined using a polyclonal apoA-I antibody. The levels of apoA-I^109−121^ and apoA-I^110−525^ were similar in the presence or absence of obstructive CAD. Among patients not using LLM, apoA-I^110−525^ adjusted for age and sex identified individuals with coronary atherosclerosis with a similar accuracy to traditional risk factors [area under the curve [AUC] (95% CI): 0.75(0.66–0.84) 0.71 (0.62–0.81)]. However, a combination of apoA-I^110−525^ with risk factors did not improve the accuracy [AUC (95% CI): 0.73 (0.64–0.82)].

**Conclusion:**

Direct two-site apoA-I assays recognizing heterogeneity in reactivity with apoA-I could provide a potential approach to identify individuals at a risk of coronary atherosclerosis. However, their clinical value remains to be studied in larger cohorts.

## Introduction

Atherosclerotic cardiovascular diseases (ASCVDs) remain as major causes of morbidity and mortality throughout the world (1). Plasma high-density lipoprotein cholesterol (HDL-C) levels are inversely associated with the risk of ASCVD (2–4). The major anti-atherogenic functions of HDL include participation in reverse cholesterol transport, and anti-inflammatory and antioxidation processes (5).

The molecular structure of HDL is complex consisting of several lipid classes and up to 85 proteins (6). In circulation, HDL consists of numerous distinct particle subpopulations varying in terms of size, charge, shape, and density (5, 7). Apolipoprotein A-I (apoA-I) is the most abundant structural protein in HDL particles constituting 60–70% of the total protein mass, with the exception of clinical conditions that harbor apoA-I genetic defects (8, 9). HDL participates in anti-atherogenic functions *via* its protein and/or lipid components (5, 10).

An emerging concept emphasizes that the functionality of HDL over its cholesterol content is reflected in the total circulating concentration of HDL. This concept is based on the failure of pharmacological interventions increasing HDL-C to reduce ASCVD events (11–13). Mendelian randomization studies also show no connection between HDL-C and the risk of coronary artery disease (CAD) or myocardial infarction (MI) (14).

Reportedly, the structural features of apoA-I may define the functional properties of HDL related to atherosclerosis and could, therefore, be explored as risk markers (10, 15). For example, plasma myeloperoxidase (MPO)-modified apoA-I (15) and lysine glycated apoA-I (16) have been shown to have an altered conformation (16). This can generate dysfunctional and pro-atherogenic apoA-I and HDL due to an impaired anti-inflammatory function and a reverse cholesterol transport function; both of which could partly account for an increased risk of CVD (15, 16).

In addition, there is a lack of monoclonal antibodies (mAbs) that could specifically target HDL functionality and could be used to improve the risk estimation of ASCVD. The production of mAbs through conventional animal immunization against a complex molecule like HDL is challenging. The phage display-based universal recombinant antibody libraries provide a platform where antibodies can be produced against almost any target (17). Recently, Huang et al. (15) demonstrated the production of an antibody against dysfunctional MPO-modified apoA-I using phage display.

We have previously developed phage-based two-site apoA-I assays (assay 109–121 and assay 110–525) by using phage-displayed single-chain variable fragment (scFv) antibodies (18) isolated against HDL derived from patients with CAD (19). However, the phage-based two-site assay design (18) was complex due to the complicated structure of the scFv antibodies (fused to a large phage particle) and the need for an additional phage detecting antibody. The aim of this study was to construct simpler forms of the two-site apoA-I assays and evaluate their clinical performance in patients undergoing coronary computed tomography angiography (CTA) and positron emission tomography (PET) myocardial perfusion imaging due to a suspected obstructive CAD. Coronary CTA is a non-invasive imaging modality that accurately detects coronary atherosclerosis and is powerful in ruling out obstructive CAD (20). In turn, PET myocardial perfusion imaging is a functional imaging modality detecting myocardial ischemia caused by obstructive CAD (20, 21). A combination of coronary CTA and PET perfusion imaging provides a comprehensive characterization of CAD, because both the extent of non-obstructive atherosclerosis and the presence of significant obstructive lesions can be evaluated (22). In this study, direct two-site apoA-I assays (assays 109–121 and 110–525) were designed using different single-chain (sc) apoA-I antibodies (sc 109, sc 121, sc 110, and sc 525) as scFv fused to bacterial alkaline phosphatase (scFv-APs). The antibodies were biotinylated or directly labeled and implemented in assay development to capture and identify the apoA-I on HDL by binding at two different sites. Therefore, these assays are referred to as direct two-site apoA-I assays.

## Materials and methods

### Clinical samples

**Figure 1:**
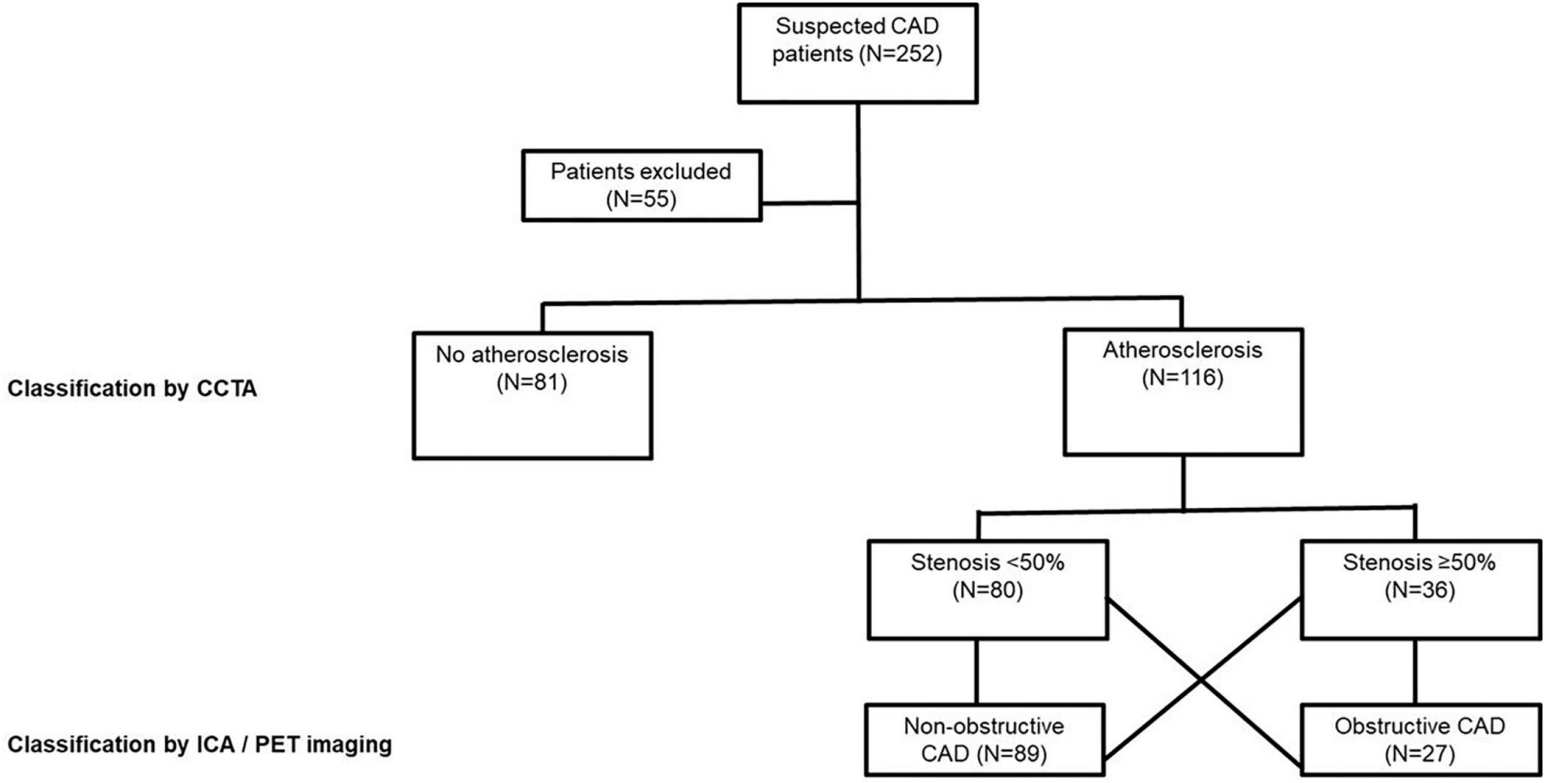
Study population. Obstructive CAD is defined as a coronary atherosclerosis associated with an abnormal stress myocardial blood flow (MBF) on a CCTA or >70% coronary stenosis on an ICA. N, number of individuals; CAD, coronary artery disease; CCTA, coronary computed tomography angiography (CCTA); PET, positron emission tomography; ICA, invasive coronary angiography.

We prospectively recruited 252 symptomatic patients referred to Turku University Hospital for coronary CTA due to suspected obstructive CAD from March 2016 to January 2019 (Figure 1). Blood samples were drawn from these patients before imaging and serum samples were prepared according to standard protocol. All the samples were stored at −70°C prior to use. The study protocols for the collection of blood samples were approved by the Local Ethics Committee of the Hospital District of Southwest Finland, and the study was conducted in accordance with the Declaration of Helsinki as revised in 2013. All samples were collected after the participants gave their informed consent. A total of 55 patients were excluded from this study due to a hemolyzed blood sample (*N* = 21), missing information on the use of lipid-lowering medication (LLM; *N* = 16), a failed or non-diagnostic coronary CTA (*N* = 11), or indications of coronary CTA other than an evaluation of a suspected CAD (*N* = 7). Hence, 197 samples remained for clinical evaluation of the direct two-site apoA-I assays.

The clinical characteristics of the patients and findings of invasive coronary angiography (ICA) were collected from electronic medical records. The Framingham Risk Score (FRS) was utilized in the calculation of a 10-year coronary heart disease (CHD) risk (23). As predictors, the FRS uses age, diabetes and smoking status, blood pressure, total cholesterol (TC), HDL-C, and low-density lipoprotein cholesterol (LDL-C).

Serum samples of randomly selected and routinely analyzed non-cardiac patients from Helsinki University Hospital (Finland) were also collected (*N* = 200). Some of these samples were used as assay controls during the optimization and validation of the direct two-site apoA-I assays.

#### Coronary CTA and PET image acquisition

Coronary CTA was performed with a 128-row hybrid PET-CT scanner (GE Discovery D690 or MI, General Electric Medical Systems, Waukesha, WI, USA), as previously described (24, 25). Before the coronary CTA, metoprolol (0–30 mg) was given intravenously to achieve a target heart rate of <60 beats/min, and an isosorbide dinitrate aerosol (1.25 mg) was administered. A coronary artery calcium scan was performed before the coronary CTA. The coronary CTA was performed using an intravenously administered low-osmolality iodine contrast agent (60–80 ml; 320–400 mg iodine/ml; injection velocity of 4–5 ml/s) followed by a saline flush. A prospectively triggered acquisition was applied whenever feasible.

As previously described (25), in the routine clinical practice at Turku University Hospital, patients initially undergo a coronary CTA using a hybrid PET-CT scanner. Immediately after the coronary CTA, the attending physician makes an initial assessment of the CTA scan to decide whether a PET myocardial perfusion imaging study is needed. Briefly, if the obstructive CAD is excluded by coronary CTA, no further imaging procedure is performed; however, in the presence of a suspected obstructive coronary lesion on the CTA (≥50% diameter stenosis), a PET myocardial perfusion imaging is performed. The PET myocardial perfusion imaging uses a ^15^O-water tracer during adenosine stress to assess the hemodynamic significance of the stenosis. In our cohort, 47 patients underwent a PET perfusion study, whereas eight patients with an obstructive CAD on the CTA were directly referred to an ICA due either to a contra-indication to the PET perfusion study (*N* = 5) or to logistic reasons (*N* = 3).

Scans were performed after an overnight fast. Patients were instructed to abstain from alcohol and caffeine for 24 h before the PET myocardial perfusion study. The adenosine infusion was started 2 min before the stress PET scan and continued at a rate of 140 μg/kg/min until the scan was complete. ^15^O-water (Radiowater Generator, Hidex Oy, Turku, Finland) was injected as an intravenous bolus (injected activity of 500–600 MBq) over 15 s, and a dynamic PET acquisition was performed (14 × 5 s, 3 × 10 s, 3 × 20 s, and 4 × 30 s).

#### Image analysis and interpretation

The coronary CTA images were analyzed according to the 17-segment vessel system using the GE ADW 4.4 Workstation software (General Electric Medical Systems, Waukesha, Wisconsin). The presence of coronary atherosclerosis and the diameter of the stenosis were evaluated in all segments. An Agatston coronary artery calcium score was measured in the non-enhanced scan.

The PET data were analyzed quantitatively using the Carimas software (developed at Turku PET Centre, Turku, Finland) (21, 26). Absolute stress myocardial blood flow (MBF) was quantified (in ml/g/min) individually for each of the standard 17 myocardial segments. Stress MBF of <2.4 ml/g/min was considered abnormally low, reflecting hemodynamically significant obstructive CAD based on our previous validation study (21). The analysis was performed by an experienced physician and recorded in a standardized reporting system.

In this study, obstructive CAD was defined as the presence of atherosclerosis on coronary CTA accompanied by an abnormally stressed MBF or, alternatively, the presence of > 70% stenosis on the ICA. The rest of the patients were classified as having a non-obstructive CAD or normal coronary arteries (no atherosclerosis) based on the CTA findings.

### Reagents

The total HDL (= HDL_2_ + HDL_3_ subclasses) from the serum of a healthy individual was isolated, as described earlier (19). Affinity-purified scFv-APs (sc 109 and sc 110) were biotinylated with 20-fold molar excess of EZ-Link-NHS-PEG4-Biotin (Thermo Scientific, USA) according to the manufacturer's instructions. Europium (Eu^+3^) chelate of tetra-tert-butyl 2,2′,2″,2^‴^-[((((4-((4-aminophenyl)ethynyl)-pyridine-2,6-diyl)bis(methylene))bis((2-(tert-butoxy)-2-oxoethyl) azanediyl))bis(ethane-2,1-diyl))bis(azanetriyl)]-tetraacetate (referred to as Eu^+3^-WN) was synthesized according to Wang et al. (27). Affinity-purified scFv-APs (sc 121 and sc 525) were labeled with 25- and 50-fold molar excess of Eu^+3^-WN, respectively, in phosphate-buffered saline (PBS; pH 7.4) at 4°C, overnight with shaking using a modified protocol of Eriksson et al. (28). Wash buffer, streptavidin-coated plates (low fluorescence and bovine serum albumin (BSA) blocked), and europium fluorescence intensifier (EFI) were purchased from Kaivogen, Finland. The HDL assay buffer contained 50 mM Tris–hydrochloride, pH 7.75, 150 mM sodium chloride, 0.05% sodium azide, 20 μM diethylenetriaminepentaacetic acid (DTPA), 20 μg/ml cherry red, 0.05% bovine γ-globulin, and 2.3% or 4% BSA.

### Measurement of biochemical parameters in clinical samples

In the samples of the CAD-suspected individuals, the biochemical parameters were measured including HDL-C, apoA-I, phospholipids (PLs), triglycerides (TG), TC, PL transfer protein (PLTP) activity, paraoxonase activity (PON-I), and serum-free glycerol. TG and TC were measured using enzymatic methods (Roche Diagnostics, Germany). HDL-C was analyzed using a phosphotungstate-MgCl_2_-precipitation method, whereby apoB-containing lipoproteins were precipitated and HDL-C could be analyzed from the supernatant after light centrifugation (29). ApoA-I was measured using a polyclonal anti-apoA-I-based ELISA instrument, as described earlier (30). Briefly, the wells were coated with a polyclonal rabbit antibody against human apoA-I, and the bound protein was detected with a horseradish peroxidase (HRP)-conjugated rabbit anti-human apoA-I immunoglobulin G (IgG) (30). LDL-C was calculated using the Friedewald formula, where LDL-C = TC-(TG/2.2)-HDL-C. PLTP activity was determined with a radiometric method as described by Jauhiainen et al. (31). PON-1 activity was measured with a chromogenic method (32). The serum concentration of free glycerol was determined by a commercial enzymatic colorimetric assay (free glycerol FS; DiaSys, Diagnostic Systems GmbH, Holzheim, Germany). Serum PL [choline-containing PL, i.e., phosphatidylcholine (PC), lysoPC, and sphingomyelin] was analyzed using the Phospholipids B Kit (Wako Chemicals, Osaka, Japan) or Pureauto S PL-Kit (Daiichi Pure Chemicals, Tokyo, Japan).

### ScFv-APs antibodies for direct two-site apoA-I assays

Two distinct pairs of recombinant scFv apoA-I antibodies (sc 109–121 and sc 110–525), which were previously tested with an optimized phage-based two-site apoA-I immunoassay and had shown diagnostic/predictive value for MI and mortality (18), were used for the development of the direct two-site apoA-I assay. The assay utilized recombinant apoA-I antibodies as scFv-AP fusion proteins, which were produced and purified as described previously (19). Briefly, DNA encoded apoA-I scFv antibodies were inserted at the Sfi-I restriction site of the chloramphenicol-resistant vector, then expressed into vector pLK06H, and purified by Ni-NTA chromatography. The purified scFv-APs were stored at 4°C in PBS.

### Final optimized direct two-site apoA-I-assays

**Figure 2:**
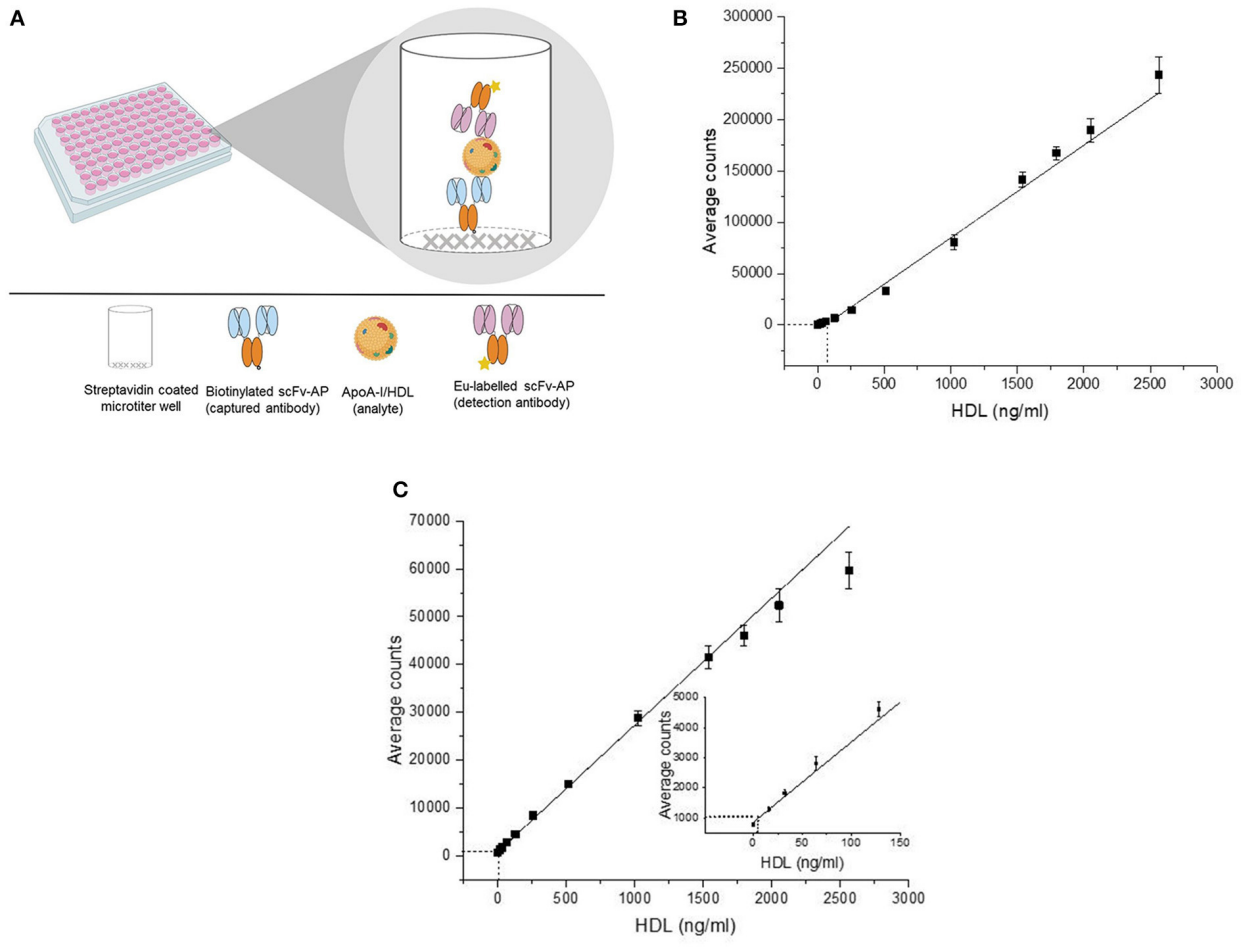
Schematic representation of the principle of the direct two-site apoA-I immunoassay **(A)** and their standard curve **(B,C)**. The detection of the direct two-site apoA-I immunoassays is based on time-resolved fluorescence of europium attached to single-chain variable fragment-alkaline phosphatase fusion protein (scFv-AP) **(A)**. In the standard curve **(B,C)**, the X-axis represents the concentration of the standard (HDL) and the Y-axis represents the average of the counts obtained from the replicates of the standard. The error bars represent the standard deviation (SD) of the average counts. The curve was fitted with a linear fitting function. The analytical sensitivity (background + 5*SD) of the assay 109–121 (63.8 ng/ml) **(B)** and assay 110–525 (6.8 ng/ml) **(C)** is illustrated as dash-line (———). ApoA-I, apolipoprotein A-I; HDL, high-density lipoprotein; Eu, europium.

The final optimized assays used the scFv-AP apoA-I antibody pairs 109–121 and 110–525. A schematic representation of the principle of these direct two-site apoA-I assays is shown in Figure 2A. Each assay used two different apoA-I antibodies, namely, the capture antibodies (scFv-AP) sc 109 and sc 110 and the corresponding Eu^+3^-WN labeled detection antibodies (scFv-AP) sc 121 and sc 525, respectively. Assay 109–121 was done with an HDL-assay buffer supplemented with 4% BSA and assay 110–525 was performed with an HDL-assay buffer containing 2.3% BSA on streptavidin plates. The total HDL (16–2,564 ng/ml as HDL total protein), i.e., HDL_2_ + HDL_3_ subpopulations isolated from the serum of a healthy individual was used as an assay calibrator or standard.

Biotinylated-captured antibodies sc 109 and sc 110 (100 ng/50 μl/well) were immobilized on separate streptavidin-coated microtiter plates and incubated for 1 h at room temperature (RT) with shaking followed by two washes. For both assays, a calibrator and sample were added in four replicates and incubated for 1 h at RT with shaking. After two washes, the Eu^+3^-WN labeled detection antibodies sc 121 (100 ng/50 μl/well) and sc 525 (200 ng/50 μl/well) were added to the respective assays and incubated for 1 h at RT with shaking. Finally, the wells were washed four times. EFI (200 μl/well) was added to the well, incubated for 10 min with shaking, and the TRF of the europium was measured with a Victor plate reader (PerkinElmer, USA).

### Assay performance and clinical evaluation

The analytical sensitivity of the direct two-site apoA-I assays was evaluated by running eleven levels of the standard (HDL protein range: 16–2,564 ng/ml) and the blanks (HDL = 0 ng/ml) through several replicates, i.e., *N* = 15 for the blanks and *N* = 12 per level for the standard in assays 109–121, and *N* = 20 for the blanks and *N* = 16 per level for the standard in assay 110–525. The analytical sensitivity was calculated by adding five times the standard deviation (SD) to the average count for the blank sample (blank + 5^*^SD) and performing a linear fitting between the concentration of the calibrator and the corresponding average counts. Within 3 days, the inter-assay variation of the assays was tested with eight routinely analyzed serum samples (HDL-C ranging from 32.5 to 49.5 mg/dl, i.e., 0.84–1.28 mmol/L), using four replicates (N) per sample, i.e., *N* = 12 per sample. The linearity of the assay was assessed by diluting the four serum samples (HDL-C ranging from 38.67 to 70.38 mg/dl, i.e., 1.0–1.8 mmol/L) from 62.5- to 1,000-fold, using three replicates per sample (*N* = 3).

The clinical performance of the optimized direct two-site apoA-I immunoassays was evaluated using serum samples from the cohort of patients with suspected obstructive CAD (*N* = 197). Comparisons were done between the following groups (Figure 1) that were categorized based on imaging findings: (i) patients without any coronary atherosclerosis (normal) vs. those with atherosclerosis (i.e., either non-obstructive or obstructive CAD), (ii) patients without obstructive CAD (i.e., either normal coronary arteries or non-obstructive CAD) vs. those with obstructive CAD, and (iii) patients without any coronary atherosclerosis (normal) vs. those with non-obstructive CAD vs. those with obstructive CAD.

### Statistical analysis

The statistical analysis was done using Origin 2015 (OriginLab Corporation, Wellesley Hills, USA), JMP Pro 13 (SAS Institute Inc., Cary, NC, USA), and IBM^®^ SPSS^®^ Statistics version 25 (IBM Corp., Armonk, NY). The normality distribution was checked with a Shapiro–Wilk test and a Q-Q plot. Categorical variables are presented as numbers (percentage) and analyzed by a chi-squared test or a Fisher's exact test, as appropriate. Continuous variables are presented as a median (25th percentile; 75th percentile). The concentration of the apoA-I obtained from the direct two-site apoA-I assays and the concentration of biochemical parameters were used after a natural log transformation was performed in order to enable parametric statistical testing.

A suitable Pearson's or Spearman's correlation was used to test the correlation of apoA-I^110−525^ or apoA-I^109−121^ levels with age, with the Agatston coronary calcium score and with conventional apoA-I ELISA (done by using polyclonal anti-apoA-I antibody). Biochemical parameters and the FRS for the CHD were compared between the two groups (no atherosclerosis vs. atherosclerosis and obstructive CAD vs. non-obstructive atherosclerosis) by using an independent *t*-test. For a comparison between the three groups (no atherosclerosis, non-obstructive CAD, and obstructive CAD), a one-way ANOVA was used followed, if required, by a multiple pair comparison using a Tukey's test.

A logistic regression analysis was performed to estimate the odds ratios (ORs) and 95% confidence intervals (CIs), with atherosclerosis and obstructive CAD as the dependent variables, and the apoA-I^110−525^ or apoA-I^109−121^ levels combined with age and gender (Model 1) or age, gender, smoking, diabetes, hypertension, LDL-C, and HDL-C (Model 2) as the independent variables. The apoA-I concentrations obtained from the two-site apoA-I assays were used as a categorical variable (below and above the median). An analysis of the area under the receiving-operating characteristic (ROC) curve was performed to test the diagnostic clinical value of the apoA-I^110−525^ or apoA-I^109−121^ levels and the FRS CHD. The ROC curves were compared using the DeLong's method. The results were analyzed for the whole cohort as well as separately in patients using and not using LLM. All the test results were considered statistically significant for *P*-value of < 0.05.

## Results

### Assay validation

The standard curves of the direct two-site apoA-I assays where the X-axis represents the HDL protein concentration (standard or calibrator) and the Y-axis represents the average counts (signal) of the standard are shown in Figures 2B,C, respectively. The measurement range of assay 109–121 and assay 110–525 was decided to be between an HDL (protein) concentration of 128–2,564 ng/ml (*r*^2^ = 0.98) and 6.8–2,564 ng/ml (*r*^2^ = 0.99). The inter-assay variation within 3 days with assay 109–121 was between 2–9% and 4–16% in the standards and samples; the inter-assay variation with apoA-I assay 110-525 in the standards and samples was 3–22% and 7–26%, respectively (Supplementary Table S1). The intra-assay variation was between 0.7–6.6% and 2.4–18.4% with assays 109–121 and 110–525, respectively. Sample dilution of 62.5- to 1,000-fold (*r*^2^ = 0.99) and 250- to 1,000-fold (*r*^2^ = 0.99) was suitable for assays 109–121 and 110–525, respectively.

### Clinical evaluation of assays

#### Characteristics of the patients

The median age of the 197 patients was 63 years and 43% were males. The most common symptom was atypical chest pain (52%). The baseline characteristics of the patients are displayed in Table 1. At the time of enrollment in the study, 72 (36.5%) patients were taking LLM. The patients taking LLM were significantly older, had more risk factors for cardiovascular disease, and were more often taking antithrombotic medication than patients without LLM. Individuals who were not using LLM had significantly higher levels of serum TC, PL, and LDL-C than those who were using LLM; however, the concentration of HDL-C, apoA-I (determined with ELISA), and other biochemical parameters was similar (Table 1).

**Table 1:** Characteristics of the study subjects.

	**All patients (*****N =*** **197)**		**LLM users (*****N =*** **72)**		**Non-LLM users (*****N =*** **125)**		
	**Data**	***N* (%)**	**Data**	***N* (%)**	**Data**	***N* (%)**	***P***
	**avaliable (*N*)**	**or median**	**avaliable (*N*)**	**or median**	**avaliable (*N*)**	**or median**	
**Age (years)**	197	63 (55–71)	72	66 (59.1–71)	125	62 (1)	0.002
**Male**	197	85 (43.2%)	72	28 (38.9%)	125	57 (45.6%)	0.37
**BMI**	132	28.3 (25–31.5)	43	27.1 (25.7–32.4)	83	28.9 (24.6–31.1)	0.80
**Risk factors**							
Smoking	180	29 (16.1%)	67	10 (14.9%)	113	19 (16.8%)	0.83
Diabetes	176	29 (16.5%)	65	18 (27.7%)	111	11 (9.9%)	0.003
Hypertension	175	107 (61.1%)	68	42 (61.8%)		65 (60.7%)	1.00
Dyslipidemia	173	93 (53.8%)	66	52 (78.8%)		41 (38.3%)	<0.001
CAD family history	160	83 (51.9%)	59	31 (52.5%)	101	52 (51.5%)	1.00
**Chest pain**	193				0.35		
Atypical	100 (51.8%)	72	40 (69%)	102	60 (58.8%)		
Typical	33 (17.1%)		14 (24.1%)		19 (18.6%)		
**FRS CHD**	191	18 (13–27)	71	17 (13–24)	120	18 (13.25–27)	0.47
**Positive exercise ECG**	118	44 (37.2%)	47	16 (34%)	71	28 (39.4%)	0.83
**LVEF**	99		36		63	0.70	
Normal (≥50%)	86 (86.9%)		32 (88.9%)		54 (85.7%)		
**Medication**							
Beta–blocker	194	74 (38.1%)	72	35 (48.6%)	122	39 (32%)	0.02
Platelet inhibitor	195	53 (27.2%)	72	38 (52.8%)	123	15 (12.2%)	0.001
Anticoagulant	193	17 (8.8%)	71	7 (9.9%)	122	10 (8.2%)	0.79
Long–acting nitrate	194	20 (10.3%)	72	15 (20.8%)	122	5 (4.1%)	0.004
Diuretic	193	43 (22.3%)	71	14 (19.7%)	122	29 (23.8%)	0.59
ACE inhibitor or ARB	197	85 (43.15%)	72	35 (48.6%)	125	50 (40%)	0.29
CCB	193	26 (13.5%)	71	10 (14.1%)	122	16 (13.1%)	0.83
Antiarrythmic	193	5 (2.6%)	71	3 (4.2%)	122	2 (16.6%)	0.35
**Biochemical parameters**							
TC (mmol/L)	197	5.2 (4.5–6.1)	72	4.7 (4.3–5.3)	125	5.5 (4.9–6.4)	<0.0001
HDL–C (mmol/L)	197	0.91(0.78–1.06)	72	0.96(0.80–1.13)	125	0.89(0.78–1.04)	0.48
TG (mmol/L)	197	1.4 (1–1.9)	72	1.4 (1, 2)	125	1.4 (1–1.8)	0.66
PL (mmol/L)	197	2.4 (2.1–2.7)	72	2.3 (2–2.5)	125	2.5 (2.2–2.8)	0.005
ApoA–I (mg/dL)	197	128 (110–147.5)	72	131 (112.3–146)	125	123 (109.5–149)	0.71
LDL–C (mmol/L)	197	3.7 (3–4.5)	72	3.1 (2.6–3.9)	125	4 (3.3–4.9)	<0.0001
Free glycerol (mmol/L)	197	0.2 (0.2–0.3)	72	0.2 (0.2–0.3)	125	0.2 (0.2–0.3)	0.86
PON–I (umol/min)	197	10 (7.4–30.5)	72	10.2 (7.6–31.5)	125	9.6 (7.1–28.6)	0.41
PLTP (nmol/ml/h)	197	6,624 (5,528–7,980)	72	6,510 (5,592.2–7,503)	125	6,741 (5,486.5–8,348)	0.1

The table summarizes the data as the median (25–75th percentile) for the continuous variables and as a percentage for the categorical variables from all the patients together (LLM and non-LLM users) and separately for those patients using LLM (LLM users) and those not using LLM (non-LLM users). The characteristics of the patients using LLM and the non-LLM users were compared. Naturally, log transformed values of all the lipid parameters and other biochemical parameters were used for statistical testing. Categorical variables were compared using Fisher's test and continuous variables were compared using a t-test.

N, number of patients; BMI, basal metabolic index; CAD, coronary artery disease; ECG, electrocardiogram; LVEF, left ventricular ejection fraction; ACE, Angiotensin-converting enzyme; ARB, Angiotensin receptor blocker; CCB, calcium channel blocker; FRS CHD, Framingham risk score for coronary heart disease in 10 years; LLM, lipid-lowering medication; apoA-I, apolipoprotein A-I; TC, total cholesterol; HDL-C, High-density lipoprotein cholesterol; TG, triglyceride; PL, phospholipids; PON-I, paraoxonase I; PLTP, phospholipid transfer protein; LDL-C, low-density lipoprotein cholesterol.

Biochemical parameters and FRS were compared between patients with and without coronary atherosclerosis (Table 2A) and between patients with and without obstructive CAD (Table 2B). Among the biochemical parameters, PL (in all patients altogether, *P* = 0.007) and PON-I (in patients not using LLM, *P* = 0.04) were higher in patients without any coronary atherosclerosis than in those with atherosclerosis (Table 2A). In the whole cohort, TC (*P* = 0.01) and LDL-C (*P* = 0.01) were higher in patients without obstructive CAD than in those with obstructive CAD (Table 2B). Among individuals not using LLM and using LLM, HDL-C (*P* = 0.03) and PLTP (*P* = 0.04) were significantly lower in patients with obstructive CAD than in those without obstructive CAD (Table 2B). Finally, FRS was significantly higher (*P* < 0.0001) in patients with coronary atherosclerosis than in those with normal coronary arteries (Table 2A), but there was no significant difference between patients with and without obstructive CAD (Table 2B).

**Table 2:** Comparison of the direct two-site apoA-I assays (assay 109–121 and assay 110–525), biochemical parameters, and FRS CHD between patients with and without atherosclerosis **(A)**, and with and without obstructive CAD **(B)**.

**(A)**									
**Parameters**	**All patients (Non-LLM and LLM users)**			**Non-LLM users**			**LLM users**		
	**No atherosclerosis** ** (*N =* 81)**	**Atherosclerosis** ** (*N =* 116)**	***P***	**No atherosclerosis** ** (*N =* 64)**	**Atherosclerosis** ** (*N =* 61)**	***P***	**No atherosclerosis** ** (*N =* 17)**	**Atherosclerosis** ** (*N =* 55)**	***P***
ApoA-I^109−121^ (mg/dl)	40.5 (30.42–53.2)	37.38 (27.87–47.45)	0.16	39.52 (29.34–54.38)	38.78 (28.7–48.5)	0.88	46.17 (33.86–53.2)	33.66 (27.74–44.21)	0.027
ApoA-I^110−525^ (mg/dl)	18.23 (14.32–26.24)	19.91 (14.66–25.99)	0.29	17.66 (13.38–24.48)	21.88 (15.89–27.44)	0.01	22.58 (17.97–31.21)	18.06 (14.4–23.51)	0.052
TC (mmol/L)	5.32 (4.58–6.23)	5.07 (4.35–5.99)	0.07	5.52 (4.83–6.34)	5.48 (4.76–6.46)	0.92	4.98 (4.36–5.55)	4.62 (4.07–5.17)	0.252
HDL-C (mmol/L)	0.94(0.78–1.01)	0.91(0.78–1.1)	0.29	0.91(0.77–1.1)	0.88(0.78–1.02)	0.58	0.96(0.87–1.23)	0.94(0.76–1.1)	0.178
TG (mmol/L)	1.33 (0.99–1.75)	1.37 (0.96–1.87)	0.71	1.3 (0.97–1.74)	1.35 (0.94–1.82)	0.82	1.34 (1–1.84)	1.41 (0.96–1.94)	0.907
PL (mmol/L)	2.46 (2.15–2.78)	2.27 (2.04–2.55)	0.007	2.49 (2.18–2.8)	2.33 (2.12–2.67)	0.23	2.4 (2.07–2.79)	2.19 (1.95–2.43)	0.055
PON-I (umol/min)	10.5 (7.6–33.75)	9.6 (7.15–27.23)	0.06	10.9 (7.53–33.78)	8.5 (6.45–23.4)	0.043	10 (8.3–39.4)	10.2 (7.6–30.8)	0.405
PLTP (nmol/ml/h)	6,660 (5,355–7,986)	6,613 (5,629–7,994)	0.89	6,557 (5,337–8,010)	6,948 (5,629–8,649)	0.48	6,888 (5,389–8,197)	6,440 (5,629–7,336)	0.975
ApoA-I (mg/dL)	123 (109.5–150)	131 (110–144.75)	0.81	122.5 (106.75–150.5)	123 (110–145)	0.76	125 (112.5–151.5)	132 (110–145)	0.882
Free glycerol (mmol/L)	0.16 (0.13–0.23)	0.17 (0.13–0.23)	0.82	0.17 (0.13–0.25)	0.17 (0.13–0.23)	0.76	0.16 (0.12–0.22)	0.16 (0.14–0.23)	0.28
LDL–C (mmol/L)	3.86 (3.06–4.69)	3.53 (2.84–4.24)	0.08	4.04 (3.25–4.83)	3.9 (3.36–4.94)	0.80	3.13 (2.8–3.98)	3.08 (2.45–3.73)	0.320
FRS CHD	15 (11–22)	22 (14–32)	<0.0001	15 (11–24)	22 (16–32)	0.22	17 (13–21.5)	17 (11–27)	0.24
**(B)**									
**Characters**	**All patients**			**Non–LLM user**			**LLM user**		
	**Obstructive CAD**			**Obstructive CAD**			**Obstructive CAD**		
	**No (*****N** =* **170)**	**Yes (*****N** =* **27)**	***P***	**No (*****N** =* **109)**	**Yes (*****N** =* **16)**	***P***	**No (*****N** =* **61)**	**Yes (*****N** =* **11)**	***P***
ApoA-I^109−121^ (mg/dl)	39.21 (29.52–50.27)	29.7 (26.04–46.52)	0.07	39.36 (29.44–50.8)	32.8 (25.74–47.34)	0.21	38.73 (29.76–50.15)	28.67 (26.04–43.19)	0.14
ApoA-I^110−525^ (mg/dl)	19.45 (14.66–27.15)	17.61 (13.52–22.01)	0.19	18.98 (14.62–26.85)	18.14 (12.05–22.53)	0.47	20.03 (14.66–27.52)	17.61 (14.78–19.99)	0.22
TC (mmol/L)	5.27 (4.57–6.16)	4.62 (4.21–5.05)	0.01	5.57 (5.02–6.49)	4.85 (4.33–5.52)	0.06	4.83 (4.29–5.37)	4.25 (3.65–4.62)	0.13
HDL-C (mmol/L)	0.92 (0.79–1.08)	0.84 (0.76–1.03)	0.1	0.90 (0.79–1.05)	0.78 (0.70–0.93)	0.03	0.96 (0.79–1.15)	0.94 (0.79–1.04)	0.14
TG (mmol/L)	1.35 (0.98–1.79)	1.43 (0.96–1.98)	0.45	1.33 (0.96–1.77)	1.39 (0.93–1.94)	0.74	1.35 (0.98–1.89)	1.43 (0.96–1.98)	0.42
PL (mmol/L)	2.34 (2.1–2.71)	2.33 (1.95–2.55)	0.08	2.44 (2.15–2.74)	2.39 (2.09–2.62)	0.39	2.24 (2–2.49)	2.2 (1.66–2.4)	0.10
PON–I (umol/min)	10 (7.58–30.55)	10.2 (6.2–26.1)	0.47	9.6 (7.4–28.6)	9.7 (6.25–30.93)	0.91	10 (7.75–31.85)	10.2 (5.6–26.1)	0.30
PLTP (nmol/ml/h)	6,655 (5,618–8,008)	6,272 (4,620–7,952)	0.23	6,741 (5,565–8,288)	6,589 (4,446–10,142)	0.74	6,604 (5,671–7,608)	5,850 (4,620–6,795)	0.04
ApoA-I (mg/dL)	126.5 (110-148.25)	134 (110-144)	0.74	123 (110–151)	121.5 (102–139)	0.24	128 (110.5–144.5)	141 (131–172)	0.03
Free Glycerol (mmol/L)	0.16 (0.13–0.23)	0.18 (0.13–0.28)	0.38	0.17 (0.13–0.23)	0.18 (0.12–0.33)	0.44	0.16 (0.13–0.23)	0.17 (0.15–0.23)	0.61
LDL–C (mmol/L)	3.78 (3.04–4.52)	3.08 (2.64–3.68)	0.01	4.07 (3.37–4.9)	3.47 (2.91–3.85)	0.08	3.13 (2.64–3.91)	2.78 (2.15–3.08)	0.11
FRS CHD	17 (13–27)	22 (15–32)	0.1	18 (13–27)	22 (18–30.75)	0.26	17 (13–24)	22 (14–32)	0.16

In the table, the clinical groups (atherosclerosis vs. no atherosclerosis, and obstructive CAD vs. without obstructive CAD) were compared in a whole patient population (LLM users and non-LLM users) and separately in those patients who were using LLM (LLM users) or not using LLM (non-LLM users). Data are shown as a median (25–75th percentile). Naturally, log-transformed values of all the parameters except for FRS CHD were used for statistical testing. Statistical testing was done using a t-test. *P*-values <0.05 were considered statistically significant.

N, number of patients; ApoA-I^109−121^, apoA-I measured with direct two-site assay 109–121; ApoA-I^110−525^, apoA-I measured with direct two-site apoA-I assay 110–525; CAD, coronary artery disease; LLM, lipid-lowering medication; TC, total cholesterol; HDL-C, high-density lipoprotein cholesterol; TG, triglyceride; PL, phospholipids; PON-I, paraoxonase I; PLTP, phospholipid transfer protein; LDL-C, low-density lipoprotein cholesterol; FRS CHD, Framingham risk score for coronary heart disease in 10 years.

ApoA-I^110−525^ (apoA-I measured by the direct two-site apoA-I assay 110–525) did not show any significant correlation (Pearson's *r*) with a conventional ELISA-based apoA-I assay (whole patient population: r = 0.05, *P* = 0.41; patients using LLM: r = −0.12, *P* = 0.29; patients not using LLM: *r* = 0.14, *P* = 0.09; Supplementary Figures S1A–C). In contrast, apoA-I^109−121^ (apoA-I measured by the direct two-site apoA-I assay 109–121) showed a weak positive correlation (whole patient population: *r* = 0.28, *P* < 0.001; patients using LLM: r = 0.17, *P* = 0.14; patients not using LLM: r = 0.33, *P* < 0.0001; Supplementary Figures S1D–F). ApoA-I^109−121^ and apoA-I^110−525^ showed a positive correlation (Spearman's *r*) with each other (whole patient population: *r*_s_ = 0.37, *P* < 0.001; patients not using LLM: *r*_s_ = 0.39, *P* < 0.001; patients using LLM: r_s_ = 0.332, *P* = 0.0042; Supplementary Figures S1G–I).

#### Direct two-site apoA-I assays and coronary atherosclerosis

Compared to patients without coronary atherosclerosis, the level of apoA-I^110−525^ was higher in patients with coronary atherosclerosis among individuals not taking LLM (*P* = 0.01, Table 2A). However, in the whole cohort, the level of apoA-I^110−525^ was similar in patients with and without coronary atherosclerosis (*P* = 0.29), since apoA-I^110−525^ tended to be lower in the presence of atherosclerosis with a borderline significance (*P* = 0.05) among patients taking LLM.

The level of apoA-I^109−121^ was significantly higher in patients without any coronary atherosclerosis than in those with coronary atherosclerosis among LLM users (*P* = 0.03) but not in patients not taking LLM (*P* = 0.88) or in the whole cohort (*P* = 0.16; Table 2A).

Neither of the two direct apoA-I assays showed a correlation (*P* > 0.05) with the extent of coronary atherosclerosis measured by the coronary calcium score.

#### Direct two-site apoA-I assays and obstructive CAD

The levels of apoA-I^109−121^ were not significantly different between the patients with and without obstructive CAD, in the whole patient population (*P* = 0.07) and separately in LLM users (*P* = 0.14) and non-LLM users (*P* = 0.21; refer to Table 2B). Similarly, the levels of apoA-I^110−525^ were also not significantly different between these patients, in the whole patient population (*P* = 0.19) and separately in LLM users (*P* = 0.22) and non-LLM users (*P* = 0.47; refer to Table 2B).

We further compared the concentration of apoA-I^109−121^ and apoA-I^110−525^ between patients with obstructive CAD, with non-obstructive CAD, or without any coronary atherosclerosis, in the whole patient cohort (Supplementary Table S2A) and separately in patients using LLM and not using LLM (Supplementary Table S2B). Among individuals who were not using LLM, the level of apoA-I^110−525^ was higher in patients with non-obstructive CAD than in those patients without atherosclerosis (*P* = 0.01) (Supplementary Table S2B). However, there was no difference in the level of apoA-I^110−525^ between patients with non-obstructive and obstructive CAD (*P* = 0.16) or obstructive CAD and without atherosclerosis (*P* = 0.99). ApoA-I^109−121^ (all patients altogether, *P* = 0.14; patients not using LLM, *P* = 0.44; patients using LLM, *P* = 0.06) could not discriminate between any of these groups (Supplementary Table S2).

#### Direct two-site apoA-I assays and cardiovascular risk factors

The median (25–75th percentile) apoA-I^109−121^ level was higher in females than in males [females: 42.07 (33.62–56.6) mg/dl, males: 31.6 (25.18–42.63) mg/dl; *P* < 0.001; Supplementary Figure S2A]. Similarly, median apoA-I^110−525^ was significantly higher in females than in males [females: 21.04 (16.36–28.82) mg/dl, males: 17.25 (12.84–22.40) mg/dl; *P* < 0.001; Supplementary Figure S2B].

We found a weak positive correlation between age and apoA-I^109−121^ levels in the whole patient population (r = 0.22, *P* = 0.002; Figure S3A) that was driven by a positive correlation among patients not using LLM (*r* = 0.22, *P* = 0.002; Supplementary Figure S3C); however, there was no correlation among patients using LLM (*r* = 0.11, *P* = 0.31; Supplementary Figure S3B). There was also a positive correlation between age and apoA-I^110−525^ among patients not using LLM (*r* = 0.20, *P* = 0.02; Supplementary Figure S3F), but no correlation was found in the whole patient population (*r* = 0.11, *P* = 0.10; Supplementary Figure S3D) or in patients using LLM (*r* = −0.04, *P* = 0.70; Supplementary Figure S3E).

Association between the direct two-site apoA-I assays and both the presence of atherosclerosis (Table 3A; Supplementary Table S3A) and obstructive CAD (Table 3B; Supplementary Table S3B) was determined in the overall study population and separately in individuals using LLM and not using LLM using models. A simple model, referred to as Model 1, included age and gender and a complex model, referred to as Model 2, included the variables in Model 1 and known risk factors for CAD including smoking, hypertension, diabetes, as well as levels of HDL-C and LDL-C. Only the simpler model (Model 1) was evaluated when estimating the association with obstructive CAD due to the small number of patients with obstructive CAD.

**Table 3:** Multivariate logistic regression analysis of the direct two-site apoA-I assay 110–525 for the presence of atherosclerosis **(A)** and obstructive CAD **(B)** in all the patients and separately for patients taking LLM (LLM users) and not taking LLM (non-LLM users).

**A: Atherosclerosis**												
	**Model 1**						**Model 2**					
**Characteristics**	**All patients**		**Non-LLM users**		**LLM users**		**All patients**		**Non-LLM users**		**LLM users**	
	**(No- LLM and LLM users)**							**(Non-LLM and LLM users)**				
	**OR (95% CI)**	***P***	**OR (95% CI)**	***P***	**OR (95% CI)**	***P***	**OR (95% CI)**	***P***	**OR (95% CI)**	***P***	**OR (95% CI)**	***P***
Age (years)	1.07(1.04–1.11)	<0.0001	1.04 (0.97–1.11)	<0.0001	1.04 (0.97–1.11)	0.215	1.08 (1.04–1.12)	0.0003	1.08(1.03–1.13)	0.004	1.03(0.94–1.13)	0.593
Male	3.08(0.33–6.25)	0.001	4.19 (1.69–10.39)	0.0010	1.83 (0.48–6.93)	0.369	2.93 (1.27–6.74)	0.011	4.94(1.51–16.23)	0.008	2.4(0.5–11.7)	0.279
ApoA–I^110−525^ (> 19 mg/dL)	1.57(0.83–2.99)	0.161	3.02 (1.30–7.04)	0.01	0.41 (0.12–1.39)	0.153	1.63 (0.77–3.46)	0.209	3.89(1.39–10.9)	0.009	0.49(0.11–2.27)	0.357
Diabetes							2.35(0.74–7.47)	0.148	6.09(0.92–40.37)	0.061	1.02(0.18–6.04)	0.987
Hypertension							1.33(0.62–2.84)	0.47	1.49(0.51–4.36)	0.472	1.33(0.29–6.14)	0.717
Smoking							1.14(0.42–3.11)	0.805	1.59(0.41–6.15)	0.505	0.36(0.05–2.73)	0.321
LDL–C (> 3.65 mmol/L)							0.7(0.33–1.49)	0.346	1(0.34–2.97)	0.991	1.11(0.23–5.48)	0.899
HDL–C (<0.91 mmol/L)							1.52(0.72–3.2)	0.280	1.78(0.64–4.97)	0.277	3.48(0.55–22.3)	0.189
**B: Obstructive CAD**												
	**Model 1**											
**Characteristics**	**All patients (Non-LLM and LLM users)**		**Non-LLM users**		**LLM users**							
	**OR (95% CI)**	***P***	**OR (95% CI)**	***P***	**OR (95% CI)**	***P***						
Age	1.08 (1.03–1.13)	0.002	1.07 (1.02–1.13)	0.012	1.1 (1–1.21)	0.061						
Male	6.54 (2.53–18.82)	**<**0.0001	6.06 (1.7–21.60)	0.006	7.81 (1.48–41.26)	0.015						
ApoA-I^110−525^ (> 19 mg/dL)	0.72 (0.28–1.78)	0.486	0.90 (0.28–2.87)	0.86	0.55 (0.11–2.60)	0.45						

Model 1 includes age, sex, and apoA-I measured with direct two-site apoA-I assay 110–525 (apoA-I^110−525^).

Model 2 includes the parameters included in model 1, and diabetes, hypertension, smoking, LDL-C, and HDL-C.

Regression analysis with Model 2 was not possible due to the very few positive cases of obstructive perfusion defect and the considerable number of parameters. In the analysis, the decided cutoff values for apoA-I^110−525^, HDL-C, and LDL-C were the median values which were used as categorical variables. CAD, coronary artery disease; LLM, lipid-lowering medicine; CI, confidence interval; OR, Odds ratio; LDL-C, low-density lipoprotein cholesterol; HDL-C, high-density lipoprotein cholesterol; apoA-I, apolipoprotein A-I.

There were no significant associations between apoA-I^109−121^ and the presence of coronary atherosclerosis (Supplementary Table S3A) or obstructive CAD (Supplementary Table S3B) when combined with age and sex (Model 1) or age, sex, and risk factors for CAD (Model 2) in the whole study cohort. However, a high level of apoA-I^110−525^ (>19 mg/dl, i.e., higher than the median value) was significantly associated with the presence of any form of coronary atherosclerosis when combined with age and sex [Model 1, OR (95% CI): 3.02 (1.30–7.04); *P* = 0.01] as well as age, sex, and risk factors for CAD [Model 2, OR (95% CI): 3.89 (1.39–10.9); *P* = 0.009] in patients not using LLM (Table 3A). There was no significant association between obstructive CAD and apoA-I^110−525^ (Table 3B).

**Figure 3:**
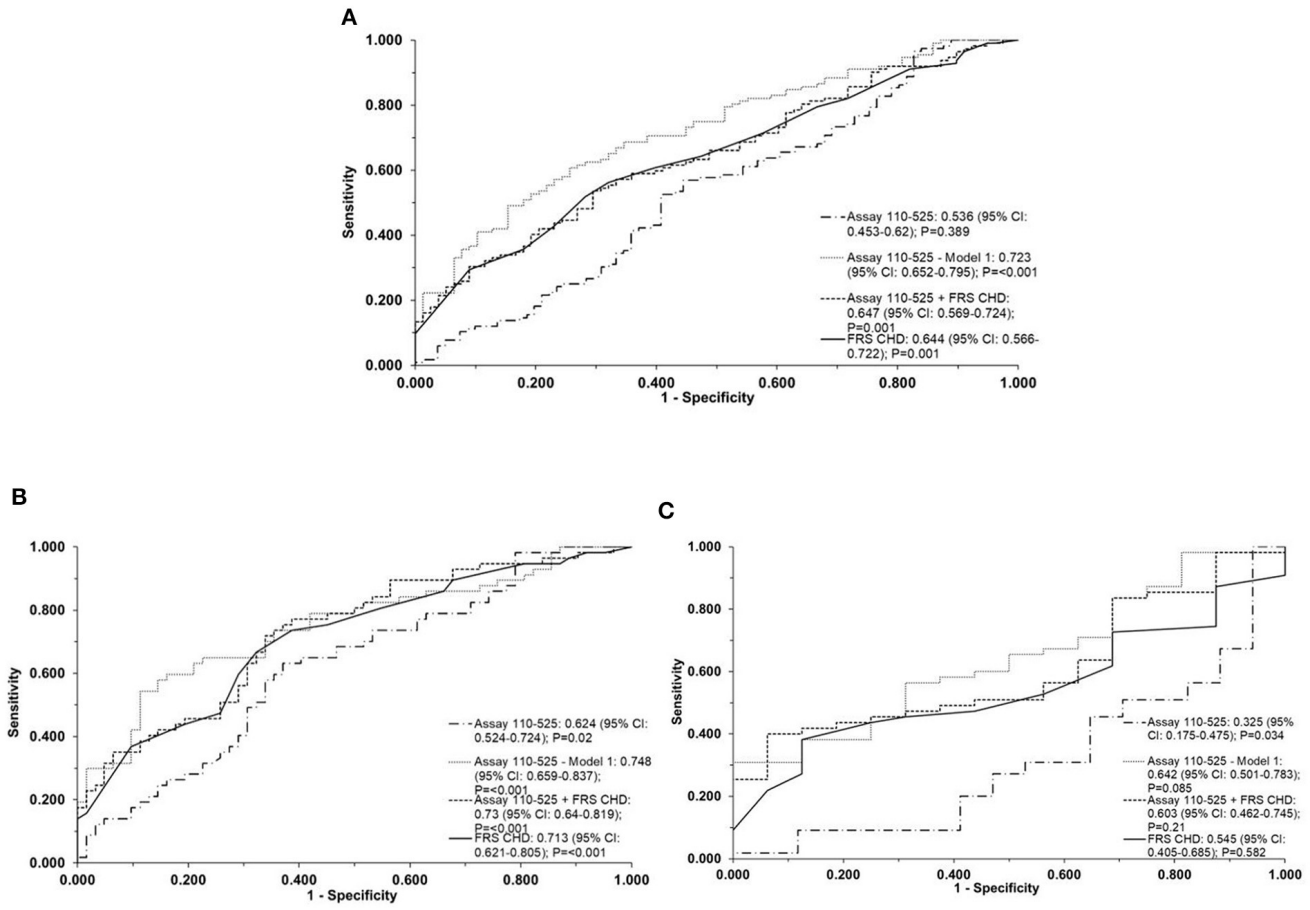
Direct two-site apoA-I assay 110-525 as predictors of coronary atherosclerosis. The ROC curve of the direct two-site apoA-I assay 110-525, assay 110-525 combined with age and sex (Model 1), direct two-site apoA-I assay 110-525 combined with a 10-year coronary heart disease risk using the Framingham Risk Score (FRS CHD), and the FRS CHD alone for detection of coronary atherosclerosis in the whole population **(A)**, in patients without LLM **(B)** and in patients on LLM **(C)**. The area under the curve (AUC) (95% CI) and the *P*-values are represented in the bottom right-hand corner of the figure. The assay 110-525, Model-1, used age and gender as the covariates. The FRS CHD estimation included age, gender, smoking, hypertension, diabetes, HDL-C, and LDL-C. ROC, receiver operator characteristic; LLM, lipid-lowering medication; HDL-C, high-density lipoprotein cholesterol; LDL-C, low-density lipoprotein cholesterol.

ApoA-I^110−525^ combined with age and sex [ApoA-I^110−525^; Model 1] provided a slightly better but not statistically significant prediction than FRS [AUC (95% CI): 0.72 (0.65–0.80) vs. 0.64 (0.57–0.72)] in the whole population, [0.64 (0.50–0.78) vs. 0.55 (0.40–0.69)] in LLM users, and [0.75 (0.66–0.84) vs. 0.71 (0.62–0.81)] in patients not using LLM (*P*-values on comparison between ROCs > 0.05) (Figure 3). ApoA-I^110−525^ combined with FRS predicted the presence of coronary atherosclerosis with a similar accuracy to FRS alone [AUC (95% CI): 0.65 (0.57–0.72) vs. 0.64 (0.57–0.72)] in the whole population, [0.60 (0.46–0.75) vs. 0.55 (0.41–0.69)] in LLM users, and [0.73 (0.64–0.82) vs. 0.71 (0.62–0.81)] in patients not using LLM (*P*-values on comparison between ROCs > 0.05) (Figure 3).

An ROC curve was not drawn for the prediction of atherosclerosis using the direct two-site apoA-I assay 109–121 since the data from this assay were not significantly associated with atherosclerosis in the logistic regression analysis (Supplementary Table S3A).

## Discussion

This study presents the development and clinical evaluation of time-resolved fluorescence (TRF) based on direct two-site apoA-I immunoassays (assays 109–121 and 110–525); the assays were established using recombinant apoA-I antibodies previously generated against the intact isolated HDL particles from plasma of patients with CAD (19). The apoA-I antibodies (sc 109, sc 121, sc 110, and sc 525) used in these direct two-site apoA-I assays were scFv fragments fused to a bacterial alkaline phosphatase (scFv-APs). In the assays, total HDL (= HDL_2_ and HDL_3_ subpopulation from normolipidemic subjects) was used as the calibrator since the apoA-I reactive epitopes of these anti-apoA-I antibodies were unknown. The optimized assays were very sensitive and a serum dilution of up to 1,000-fold was found to be sufficient for these assays. We evaluated whether differences detected by the new recombinant apoA-I antibodies and the respective direct two-site apoA-I assays are related to coronary atherosclerosis and its severity in patients with suspected obstructive CAD.

At present, LLM (mainly statins) is widely used for the prevention of cardiovascular disease by reducing LDL-C and TG (33) and its effect on HDL particles (34, 35). As these drugs also affect HDL (the effect is not systematically similar in each subject), we analyzed the data separately for patients with and without the use of LLM, in addition to the whole cohort. In line with the prescription of LLM according to the composite risk of ASCVD (36), subjects who were using LLM were older and had more risk factors for cardiovascular disease than patients not taking LLM. Subjects not using LLM displayed a higher level of TC, PL, and LDL-C, but interestingly there were no significant differences in levels of HDL-C or apoA-I determined by using the apoA-I ELISA method (30). The apoA-I level determined with the direct two-site apoA-I assay 109–121 (referred to as apoA-I^109−121^ ) and assay 110–525 (referred to as apoA-I^110−525^) was significantly higher in females compared to males. We found a positive correlation between direct two-site apoA-I assays and age specifically in patients not using LLM. The direct two-site apoA-I assays 110–525 did not show any significant correlation with a conventional ELISA-based apoA-I test; however, the assays 109–121 (in all the patients altogether and in patients without LLM) showed a weak positive correlation. It is worth noting that the ELISA-based apoA-I assay was performed using polyclonal antibodies against purified apoA-I (30). However, the direct two-site apoA-I assays use antibodies derived from the phage display library against the whole HDL particle and the epitopes of these antibodies are not yet characterized. Since these direct two-site apoA-I assays use as a calibrator HDL derived from the normolipidemic, disease-free subject, this indicates that the epitopes reacting with these mAbs also exist in HDL particles not modified by the disease itself. However, it is possible that among the different patient groups, the reacting epitopes could be sterically hindered/or induced to interact with these mAbs.

Among all the measured biochemical parameters (namely TG, TC, PL, HDL-C, LDL-C, apoA-I, PON-I, PLTP, and free glycerol), only the total PL serum and PON-I activity were significantly lower in patients with coronary atherosclerosis, which is in accordance with previous studies (37, 38). The reduced PL in HDL might reflect a reduced efflux capacity in the cholesterol from macrophage-foam cells; however, we have measured only the total serum PLs and not separately in each of the isolated HDL particles. The reduced PON-I activity indicates that anti-oxidative capacity mediated *via* the function of PON-1 is compromised in these subjects. The PON-1 function is physiologically important to attenuate the LDL oxidation generating minimally modified oxLDL which is related to atherogenesis (39). Notably, neither HDL-C nor LDL-C was significantly associated with atherosclerosis in the multivariable analyses. This finding further highlights, especially in the case of HDL, that the quantity of HDL-C alone has a limited value in the prediction of the presence of atherosclerosis. Moreover, in the case of LDL-C, evidence of the superiority of apoB-100 concentration over LDL-C in the evaluation of CHD risk is increasingly gaining strength; for instance, strong evidence has been shown in the study on the Framingham Offspring Cohort (40).

Previous studies have demonstrated that a low level of serum apoA-I is associated with CAD and MI (41), (42). Other studies have identified modified forms of apoA-I as poor acceptors of cholesterol from macrophage-foam cells during the process of reverse cholesterol transport (43–45), which is an important atheroprotective mechanism facilitated by HDL. Increased levels of modified apoA-I forms, such as chlorinated (46) and oxidized apoA-I (47), have been found in patients with CAD and acute coronary syndromes. In this study, the levels of apoA-I (determined with a conventional ELISA assay) were similar among patients with and without coronary atherosclerosis. However, a higher level of apoA-I^110−525^ was associated with coronary atherosclerosis in patients not using LLM. This association was present in multivariable models including age, gender, and traditional CAD risk factors. We also tested whether apoA-I^110−525^ could improve the identification of patients with coronary atherosclerosis as compared to traditional risk factors. FRS is a well-established method to evaluate the 10-year risk of having CHD (23). ApoA-I^110−525^ in combination with FRS predicted coronary atherosclerosis with a similar diagnostic accuracy as FRS alone. Notably, apoA-I^110−525^ adjusted for age and sex performed slightly better than FRS (based on an incremental AUC) in individuals not using LLM than in those taking LLM. Whether this approach could be used to estimate the likelihood of coronary atherosclerosis in LLM naive individuals remains to be tested in a larger cohort. In contrast to apoA-I^110−525^, apoA-I^109−121^ was lower in the presence of atherosclerosis among LLM users, indicating that these antibodies recognize different epitopes. The difference in the apoA-I^109−121^ levels was not significant when the risk factors were included.

The amount of coronary artery calcium reflects the extent of the coronary atherosclerosis and allows a risk prediction for the general population (48). The two-site apoA-I assays were not correlated/associated with the extent of coronary artery calcium or the presence of obstructive CAD (abnormal stress MBF or stenosis >70%). The lack of correlation with obstructive CAD might also be due to the small number of individuals with obstructive CAD in our study population, especially after stratification according to the use of LLM. The lack of correlation with the coronary calcium score might be explained by the fact that calcification represents a relatively late phenomenon in the progression of atherosclerosis, whereas serum lipid derangements will contribute to earlier stages of atherosclerosis. However, the coronary calcium score can be seen as an early marker of atherosclerotic disease in an individual as it shows the disease when it is in an early clinical phase. We do not have any specific explanation for our observation and this issue needs/requires further study.

There are some limitations to this study, which must be considered. Due to the small number of patients, the findings may be viewed as exploratory, but they demonstrate the proof-of-concept that recombinant apoA-I antibodies derived from the phage display library against CAD HDL particles may be used to improve the estimation of the risk of coronary atherosclerosis. However, the findings need to be confirmed in a larger cohort. With regard to the antibodies used in the direct two-site assays, they have already been characterized for their various properties as a part of the study where they were discovered and further investigated (18, 19). SDS-PAGE analysis demonstrated that the antibodies had adequate purity after affinity chromatography purification and the correct molecular size (19). Moreover, the DNA and translated polypeptide sequences of the antibodies are known. In addition, the purified biotinylated or europium chelate-labeled antibodies have been stored at +4°C for months without observed aggregates. However, any systematic study on their long-term stability in storage has unfortunately not been conducted. The antibodies used in this study and the aforementioned previous study (18) are in the form of single-chain antibody fragment (scFv) fused to bacterial alkaline phosphatase, i.e., scFv-AP. Although this construct corresponds to an IgG molecule in terms of certain relevant features such as its bivalent nature (i.e., alkaline phosphatase is a homodimer) and size (~150 kDa of scFv-AP dimer vs. 160 kDa of IgG), scFv-AP can still be a less optimal format for an immunoassay reagent than the intact IgG, which is considered a standard antibody format used in immunoassays. This can be a reason behind the somewhat high variation observed, especially with the direct two-site assay 110–525, and therefore also calls for/requires further optimization of this assay. Conversion of these antibodies to intact IgG, i.e., the format typically used in immunoassays, could enhance the robustness of these antibodies and help to make them more predictable as reagents. However, the conversion includes a risk of altering the binding properties of the antibodies, and the process should, therefore, involve careful characterization of binding properties and the possibility for additional genetic engineering of the binders. Unfortunately, this was out of the scope of this study but can be considered for future research work. In addition, these antibodies were characterized and studied for their ability to bind several different HDL forms and some HDL-associated proteins (19); however, their exact epitopes on apoA-I polypeptide are not known. Detailed characterization of the epitope responsible for the binding of antibodies as well as a functional characterization of the detected HDL particles would help to better understand the significance of the assays. For instance, dysfunctional apoA-I could be associated with an impaired ability of HDL to act as a cholesterol acceptor from macrophage-foam cells.

In summary, TRF-based direct two-site apoA-I immunoassays were developed using novel recombinant apoA-I antibody pairs (sc 109–121 and sc 110–525). The assays were clinically evaluated and compared with other traditional risk predictors (HDL-C, LDL-C, and FRS) with a well-characterized clinical cohort of patients with suspected obstructive CAD. In patients not using LLM, a high level of apoA-I identified with the antibody pair 110–525 was associated with the presence of coronary atherosclerosis; however, HDL-C and apoA-I levels measured using a polyclonal anti-apoA-I-based ELISA test were not. The direct two-site apoA-I assay 110–525 showed a similar prediction of atherosclerosis to FRS. In conclusion, assays targeting heterogeneity in HDL's main apolipoprotein, apoA-I, could provide a potential approach to improving the identification of patients with a risk of developing coronary atherosclerosis.

## Data availability statement

The raw data supporting the conclusions of this article will be made available by the authors, without undue reservation.

## Ethics statement

The studies involving human participants were reviewed and approved by Local Ethics Committee of the Hospital District of Southwest Finland. The patients/participants provided their written informed consent to participate in this study.

## Author contributions

KP, JL, UL, MJ, JK, and AS contributed to the study concept and design. PN, TH, KV, and JM performed the laboratory work. PN and AS contributed to data analysis. AS, ET, WN, TM, and JK contributed to the collection of patients' samples or medical information or image acquisition and interpretation. PN, AS, and JM wrote the first draft of the manuscript. All authors contributed to the article and approved the submitted version.

## Funding

This research was supported by the Doctoral Program of Clinical Investigation (CLIDP), University of Turku Graduate School (to PN), the Academy of Finland (grant # 257545 to MJ, grant # 310136 to AS), and the Finnish Foundation for Cardiovascular Research (to MJ and AS).

## Conflict of interest

Author AS declares fees for lectures or consultancy from Abbott, Amgen, Astra Zeneca (AS and JK), Boehringer Ingelheim, Bayer and Pfizer, Novartis (JK), GE Healthcare (JK). The remaining authors declare that the research was conducted in the absence of any commercial or financial relationships that could be construed as a potential conflict of interest.

## Publisher's note

All claims expressed in this article are solely those of the authors and do not necessarily represent those of their affiliated organizations, or those of the publisher, the editors and the reviewers. Any product that may be evaluated in this article, or claim that may be made by its manufacturer, is not guaranteed or endorsed by the publisher.
