# Supplementary material for: Time-resolved fluorescence based direct two-site apoA-I immunoassays and their clinical application in patients with suspected obstructive coronary artery disease
Source: Front Cardiovasc Med. 2022 Oct 14;9:912578. doi: 10.3389/fcvm.2022.912578 (PMC9614376; doi:10.3389/fcvm.2022.912578)
Supplement: Supplementary file 1 [file Data_Sheet_1.pdf]

Time-resolved fluorescence based direct two-site apoA-I immunoassays and their clinical application in patients with suspected obstructive coronary artery disease

Authors: Priyanka Negi[1], Taina Heikkilä[1], Karoliina Vuorenpää[1], Emilia Tuunainen[1], Wail Nammas[2,3] , Teemu Maaniitty[3] , Juhani Knuuti[3], Jari Metso[4], Janita Lövgren[1], Matti Jauhiainen[4], Urpo Lamminmäki[1], Kim Pettersson[1], Antti Saraste[2]

Affiliations

- [1]Department of Life Technologies/Biotechnology, University of Turku, Turku, Finland.
- [2]Heart Center, Turku University Hospital and University of Turku, Turku, Finland.
- [3]Turku PET Centre, Turku University Hospital and University of Turku, Turku, Finland
- [4]Minerva Foundation Institute for Medical Research, Biomedicum, Helsinki, Finland; National Institute for Health and Welfare, Genomics and Biobank Unit, Biomedicum 2U, Helsinki, Finland.

*** Correspondence:**
Priyanka Negi
priyanka.micro@gmail.com
Antti Saraste
antsaras@ utu.fi

Supplementary Tables

Table S1. Inter-assay variation of direct two-site apoA-I assays.

**Standard (HDL; ng/ml)**	**Assay 109-121 (CV%)**	**Assay 110-525 (CV%)**
0	5%	3%
16	2%	6%
32	2%	7%
64	6%	14%
128	5%	19%
256.4	7%	18%
512.9	8%	15%
1025.7	9%	21%
1538.5	5%	22%
1794.9	4%	18%
2051.3	6%	11%
2564	7%	18%
**Sample ID (HDL-C; mmol/L)**		
11 (1.28)	12%	26%
12 (1.00)	10%	13%
18 (0.92)	4%	19%
27 (0.84)	7%	13%
29 (1.04)	15%	7%
42 (1.23)	7%	22%
71 (1.23)	12%	19%
76 (1.21)	16%	17%

The assays were performed three times on three different days with replicates (n) of each sample (n=12) and the standard (HDL 0 ng/ml, n=15; HDL 16-2564 ng/ml, n=12). The CVs (%) of the average signals is presented. HDL, high density lipoprotein; HDL-C, High density lipoprotein cholesterol; CV, coefficient of variation.

Table S2: Comparison of apoA-I concentrations measured by the direct two-site apoA-I assays between patients with no atherosclerosis (no atherosclerosis), with non-obstructive CAD and with obstructive CAD in the whole cohort (A) and separately for LLM-users and non-LLM users (B).

A. Whole cohort (all patients).

**ApoA-I**	**All patients (Non-LLM and LLM users)** No atherosclerosis (N= 81)	Non-obstructive CAD (N= 89)	Obstructive CAD (N= 27)	**P**
**ApoA-I$^{109-121}$ (mg/dl)**	40.5 (30.42-53.2)	37.9 (28.98-48.5)	29.7 (26.04-46.52)	0.14
**ApoA-I$^{110-525}$ (mg/dl))**	18.23 (14.32-26.24)	20.2 (15.49-27.34)	17.61 (13.52-22.01)	0.12

B. Patients using LLM and not using LLM.

**ApoA-I**	**Non-LLM users**				**LLM users**			
	**No atherosclerosis (N= 64)**	**Non-obstructive CAD (N= 45)**	**Obstructive CAD (N= 16)**	**P**	**No atherosclerosis (N= 17)**	**Non-obstructive-CAD (N= 44)**	**Obstructive CAD (N= 11)**	**P**
**ApoA-I$^{109-121}$ (mg/dl)**	39.5 (29.3-54.4)	39.2 (29.4-50.6)	32.8 (25.7-47.3)	0.44	46.2 (33.9-53.2)	34.1 (27.9-44.4)	28.7 (26.0-43.2)	0.06
**ApoA-I$^{110-525}$ (mg/dl))**	17.7 (13.4-24.5)	22.8 (17.6-29.3)	18.1 (12.1-22.5)	0.01*	22.6 (17.9-31.2)	19.2 (14.3-26.4)	17.6 (14.9-19.9)	0.11

In the tables, data is shown as a median (25-75th percentile). Clinical groups (no atherosclerosis vs. non-obstructive CAD vs. obstructive CAD) were compared in the whole patient population (LLM users and no-LLM users) (A) and separately for LLM users and non-LLM users (B). Overall comparison between the clinical groups was done using a one-way ANOVA (P vales are shown in the table) and, if significant, a pairwise comparison was done with a Tukey's test. P values < 0.05 were considered significant. Naturally log transformed values were used for statistical testing. * $P < 0.05$ for comparison between no atherosclerosis and non-obstructive CAD.

N, number of patients; CAD, coronary artery disease; LLM, lipid lowering medication; ApoA-I$^{109-121}$ and ApoA-I $^{110-525}$: apolipoprotein A-I measured by direct two-site apoA-I assay 109-121 and 110-525, respectively.

Table S3. Multivariate logistic regression analysis of the direct two-site apoA-I assay 109-121 for the presence of atherosclerosis (A) and obstructive CAD (B) in all the patients and separately in LLM users and non-LLM users.

A. Atherosclerosis

	**Model 1**						**Model 2**					
**Characteristics**	**All patients (Non-LLM and LLM users)**		**Non-LLM users**		**LLM users**		**All patients (Non-LLM and LLM users)**		**Non-LLM users**		**LLM users**	
	**OR (95% CI)**	**P**	**OR (95% CI)**	**P**	**OR (95% CI)**	**P**	**OR (95% CI)**	**P**	**OR (95% CI)**	**P**	**OR (95% CI)**	**P**
Age (years)	1.09(1.05-1.12)	<0.0001	1.09(1.05-1.13)	<0.0001	1.06(0.99-1.13)	0.13	1.09(1.04-1.13)	0.0001	1.09(1.04-1.14)	0.001	1.05(0.96-1.15)	0.37
Male	2.41(1.19-4.85)	0.01	2.92(1.24-6.9)	0.01	1.71(0.45-6.53)	0.43	2.5(1.09-5.73)	0.03	3.55(1.19-10.62)	0.02	1.86(0.37-9.41)	0.45
ApoA-I $^{109-121}$ (> 39 mg/dL)	0.58(0.3-1.11)	0.09	0.73(0.33-1.65)	0.44	0.39(0.12-1.28)	0.11	0.56(0.25-1.24)	0.14	0.68(0.25-1.91)	0.46	1.09(0.92-0.22)	0.15
Diabetes							2.78(0.86-9.04)	0.09	5.27(0.84-33.18)	0.07	0.98(0.16-5.98)	0.97
Hypertension							1.29(0.6-2.76)	0.52	1.49(0.54-4.14)	0.45	1.62(0.35-7.53)	0.54
Smoking							1.17(0.43-3.17)	0.76	1.82(0.52-6.44)	0.35	0.35(0.05-2.72)	0.31
LDL-C (> 3.65 mmol/L)							0.85(0.39-1.86)	0.68	1.16(0.39-3.43)	0.79	1.09(0.22-5.5)	0.91
HDL-C (</= 0.91 mmol/L)							1.16(0.55-2.45)	0.70	1.17(0.44-3.15)	0.76	3.53(0.59-21.35)	0.16

B. Obstructive CAD

	**Model 1**					
**Characteristics**	**All patients (Non-LLM and LLM users)**		**Non-LLM users**		**LLM users**	
	**OR (95% CI)**	**P**	**OR (95% CI)**	**P**	**OR (95% CI)**	**P**
Age (years)	1.08 (1.04-1.14)	0.002	1.07 (1.02-1.13)	0.012	1.1 (1-1.21)	0.061
Male	6.5 (2.53-18.56)	<0.0001	5.92 (1.68-20.9)	0.006	8.15 (1.53-43.43)	0.015
ApoA-I $^{109-121}$ (>39 mg/ml)	0.62 (0.24-1.57)	0.329	0.66 (0.2-2.16)	0.495	0.64 (0.13-3.14)	0.587

Model 1: includes age, sex and apoA-I measured with two site apoA-I assay 109-121(ApoA-I$^{109-121}$). Model 2: includes parameters included in model 1 and diabetes, hypertension, smoking, LDL-C and HDL-C. Regression analysis with Model 2 was not possible due to the very few defect positive cases of obstructive perfusion and the considerable number of parameters. In the analysis, the cut-off values determined for ApoA-I$^{109-121}$, HDL-C and LDL-C were the median values which were used as the categorical variable. CAD, coronary artery disease; LLM, lipid lowering medicine; CI, confidence interval; OR, Odds ratio; LDL-C, low density lipoprotein cholesterol; HDL-C, high density lipoprotein cholesterol; apoA-I, apolipoprotein A-I.

Supplementary Figures:

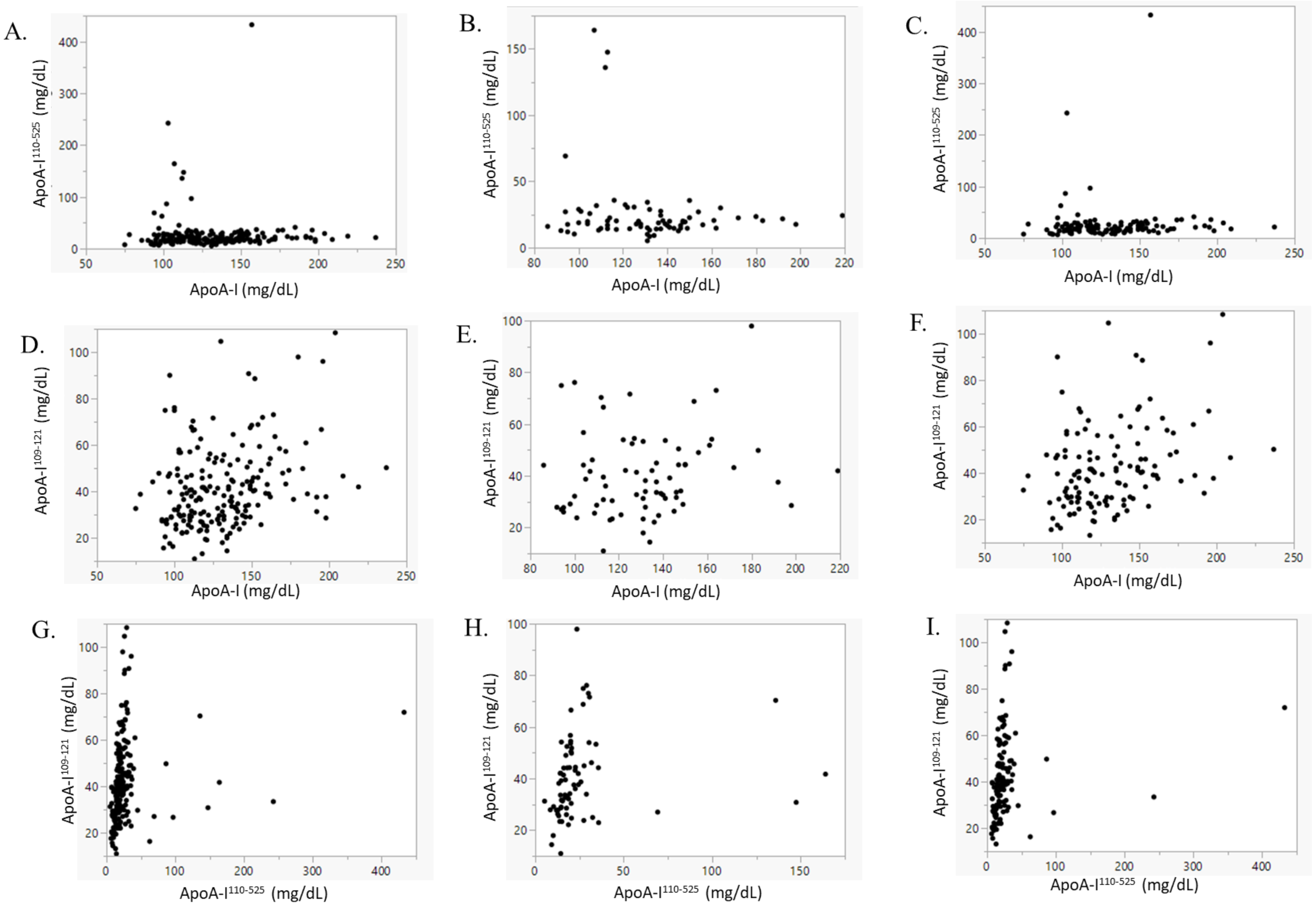

Figure S1. Correlation between the direct two-site apoA-I assays (assay 110-525 and 109-121) and the conventional apoA-I ELISA (A-F), and, between the two-site apoA-I assays (G-I) in the whole population (A, D, G) of patients suspected of obstructive CAD and separately in patients using LLM (B, E, H) and not using LLM (C, F, I). In the figures (A-F), X-axis

represents the concentration of conventional ELISA based apoA-I and Y-axis represents concentration of apoA-I obtained from direct two-site apoA-I assay 110-525 (referred apoA-I$^{110\text{-}525}$) and 109-121 (referred as apoA-I$^{109\text{-}121}$). In figures G-I, the X-axis and Y-axis represent the concentration of apoA-I$^{110\text{-}525}$ and apoA-I$^{109\text{-}121}$, respectively.

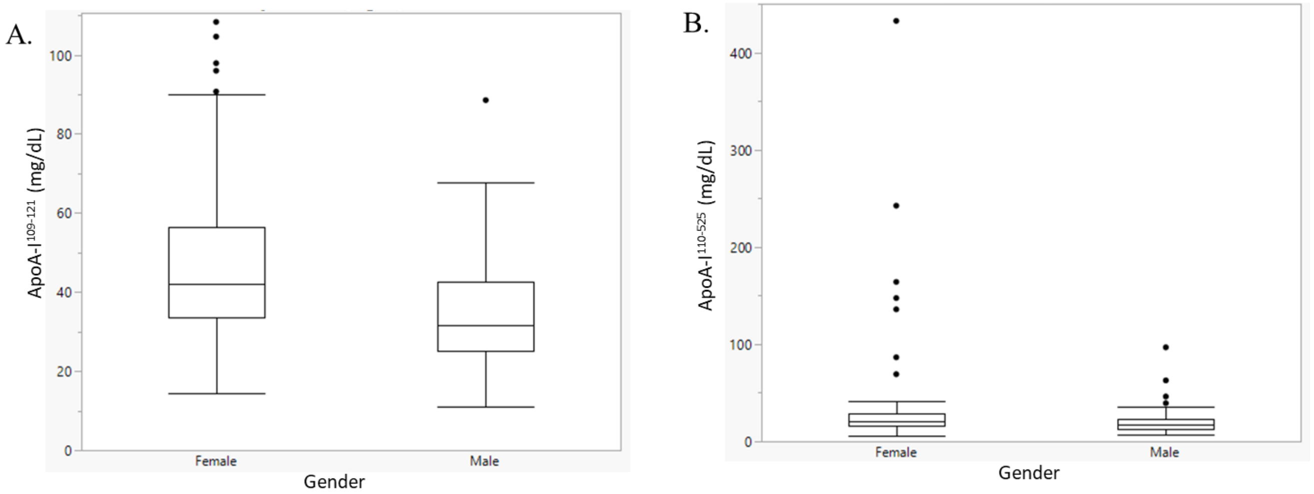

Figure S2. Box plot showing the concentration of apoA obtained with the direct two-site apoA-I assay 109-121 (referred apoA-I$^{109\text{-}121}$) and 110-525 (referred as apoA-I$^{110\text{-}525}$) in females and males suspected of obstructive CAD. The bottom and top of the box represent the 25th and 75th percentile, and, the horizontal line within the box represents the median. The bottom and top of the whiskers represent the minimum and maximum values. The solid circles (.) outside the box represents the outliers. Description of the respective panels (A, B) can be found in Table 1.

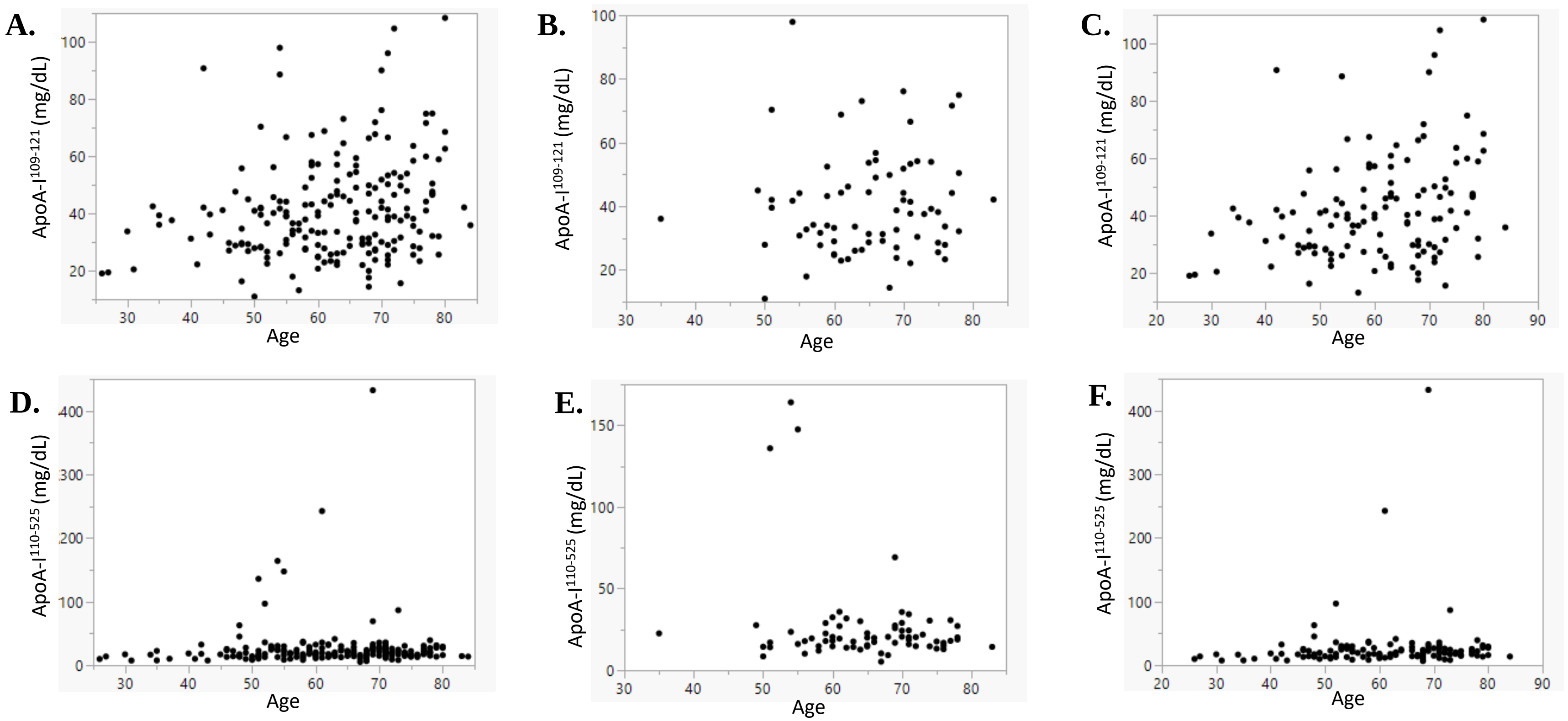

Figure S3. Correlation between the concentration of apoA-I obtained with the direct two-site apoA-I assays 109-121 (apoA-I$^{109-121}$) and 110-525 (apoA-I$^{110-525}$), and the age of the whole population of patients suspected of obstructive CAD (A, D) and separately in patients using LLM (B, E) and not using LLM (C, F). In the figures, the X-axis represents the age of the patients and Y-axis represents the concentration of apoA-I obtained from the two-site apoA-I assay 110-525 and 109-121.
